# EXPLORING PALLIATIVE CARE DISPARITIES IN RACIALLY AND ETHNICALLY DIVERSE COMMUNITY-DWELLING OLDER ADULTS

**DOI:** 10.1093/geroni/igz038.2734

**Published:** 2019-11-08

**Authors:** Daniel S Gardner, Meredith Doherty

**Affiliations:** 1 Silberman School of Social Work, Hunter College, CUNY, New York, New York, United States; 2 the Graduate Center, CUNY, New York, New York, United States

## Abstract

Despite the growth and recognized benefits of palliative care for people with serious illness and their families, there are significant racial and ethnic disparities in access to and utilization of services, particularly among older adults living in impoverished, medically-underserved communities. This paper presents preliminary findings from a mixed-method, CBPR study exploring the experiences, supportive care needs, and service use of diverse older adults living with serious illness in an urban, medically-underserved community in the U.S. Systematic analyses of focused, semi-structured interviews with 45 older adults identified cultural, environmental, financial, and structural barriers to palliative care, and identified the critical importance of familial, social, spiritual, and formal networks of support in coping with serious illness and associated symptoms. The investigators describe implications for practice and policy that addresses palliative care disparities, and strategies for engaging with communities to extend culturally-sensitive palliative care to diverse, community-dwelling older adults and their social networks.

